# Nanogels with High Loading of Anesthetic Nanocrystals for Extended Duration of Sciatic Nerve Block

**DOI:** 10.1021/acsami.1c00894

**Published:** 2021-04-06

**Authors:** Teresa Alejo, Laura Uson, Guillermo Landa, Martin Prieto, Cristina Yus Argón, Sara Garcia-Salinas, Ricardo de Miguel, Ana Rodríguez-Largo, Silvia Irusta, Victor Sebastian, Gracia Mendoza, Manuel Arruebo

**Affiliations:** † Instituto de Nanociencia y Materiales de Aragón (INMA), CSIC-Universidad de Zaragoza, Zaragoza 50009, Spain; ‡ Department of Chemical Engineering, University of Zaragoza, Campus Río Ebro—Edificio I+D, C/ Poeta Mariano Esquillor S/N, 50018 Zaragoza, Spain; § Department of Animal Pathology, Veterinary Faculty, University of Zaragoza, 50013 Zaragoza, Spain; ∥ Networking Research Center on Bioengineering, Biomaterials and Nanomedicine, CIBER-BBN, 28029 Madrid, Spain; ⊥ Aragon Health Research Institute (IIS Aragón), 50009 Zaragoza, Spain

**Keywords:** bupivacaine nanocrystals, local anesthesia, nerve blockade, drug delivery, thermoresponsive nanogels

## Abstract

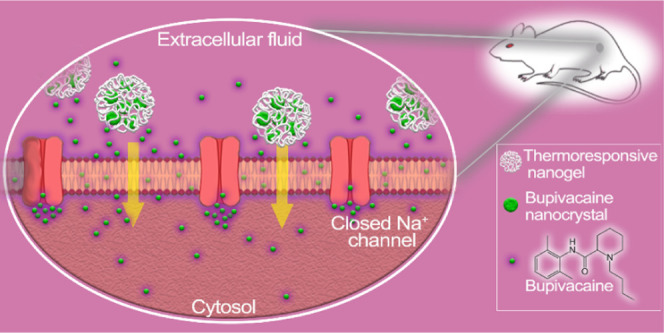

The development of thermoresponsive nanogels loaded with nanocrystals of the local anesthetic bupivacaine nanocrystals (BNCs) for prolonged peripheral nerve pain relief is reported here. BNCs were prepared using the antisolvent precipitation method from the hydrophobic form of bupivacaine (bupivacaine free base). The as-prepared BNCs were used stand-alone or encapsulated in temperature-responsive poly(ethylene glycol) methyl ether methacrylate (OEGMA)-based nanogels, resulting in bupivacaine NC-loaded nanogels (BNC-nanogels) of monodisperse size. The synthesis protocol has rendered high drug loadings (*i.e*., 93.8 ± 1.5 and 84.8 ± 1.2 wt % for the NC and BNC-nanogels, respectively) and fast drug dissolution kinetics in the resulting composite material. *In vivo* tests demonstrated the efficacy of the formulation along with an extended duration of sciatic nerve block in murine models of more than 8 h with a formulation containing only 2 mg of the local anesthetic thanks to the thermoresponsive character of the polymer, which, at body temperature, becomes hydrophobic and acts as a diffusion barrier for the encapsulated drug nanocrystals. The hydrophobicity of the encapsulated bupivacaine free base probably facilitates its pass through cell membranes and also binds strongly to their hydrophobic lipid bilayer, thereby protecting molecules from diffusion to extracellular media and to the bloodstream, reducing their clearance. When using BNC-nanogels, the duration of the anesthetic blockage lasted twice as long as compared to the effect of just BNCs or a conventional bupivacaine hydrochloride solution both containing equivalent amounts of the free drug. Results of the *in vivo* tests showed enough sensory nerve block to potentially relieve pain, but still having mobility in the limb, which enables motor function when required. The BNC-nanogels presented minimal toxicity in the *in vivo* study due to their sustained drug release and excellent biocompatibility. The encapsulation of nano-sized crystals of bupivacaine provides a prolonged regional anesthesia with reduced toxicity, which could be advantageous in the management of chronic pain.

## Introduction

Pain treatment is a clinical challenge owing to the short duration of anesthetic effects, associated with the low molecular weight of the drugs, and the potential systemic toxicity provided by current existing treatments.^1^ At present, clinical local or regional anesthetic effects rarely last beyond 12 h unless in case of using continuous catheter infusions.^2,3^ Most common prescriptions are based on antipyretic analgesics and opioids; nevertheless, these medications present severe adverse side effects, such as dizziness, nausea, vomiting, constipation, physical dependence, and respiratory complications.^4,5^ The development of an effective injectable local anesthetic with prolonged duration of action would enhance the quality of life of patients affected by chronic or postoperative pain.^6^

Conventional free drugs present several disadvantages that impair their optimal performance, such as short half-life in the organism, high dosages administered, poor selectivity, and associated side effects in healthy tissues. The incorporation of drugs into nanoparticles can improve the therapeutic characteristics of the formulations and outweigh some of those drawbacks. Drug nanoencapsulation has been demonstrated to reduce its systemic toxicity and opens up the possibility of adding targeting characteristics that enhance the accumulation of the nanomedicine at diseased sites.^7^ However, for an efficient treatment, the amount of drug loaded into the carrier nanomaterial must be high enough. Usually, nanomedicines have shown low drug-loading contents (in many cases less than 10 wt %).^8−12^ Therefore, efforts must be made to develop nanomedicines in which the ratio of payload to nanomaterial could be increased. Nanomedicines with high drug loadings (DLs) can deliver large amounts of drug per nanomaterial unit. Also, the reduction of the nanoparticulate carrier in the total nanomedicine content can decrease the overall nanomaterial fabrication cost and also reduce the potential side effects associated with the carriers themselves.^13^ Drug loading mechanisms driven by supramolecular interactions such as electrostatic and physical adsorption often outcome low drug-loading efficiencies; meanwhile, high drug loadings can be obtained in the case of crystallization and when using covalent and coordinative bonds.^14−17^ However, chemical modification of the transported drug should be avoided to preserve its therapeutic function.

Nanocrystals (NCs) have been used during the past 20 years to improve the bioavailability and dissolution rate of poorly water-soluble hydrophobic drugs in several formulations intended for oral delivery.^18,19^ Nanocrystallization is recommended for the enteral delivery of drugs belonging to the Class II Biopharmaceutics Classification System (*i.e*., high permeability and low solubility). In this way, by changing the size to the nanosize range, the specific surface area is increased, giving an enhancement in the rate of dissolution in physiological media.^20^ In fact, several Food and Drug Administration (FDA) approved nanocrystal-based drugs are already available on the market.^18^ Those commercially available products are usually prepared by top–down methods, including media milling and high-pressure homogenization.^21,22^ The number of FDA-approved products based on NCs prepared by bottom-up methods is very limited, although they have been demonstrated to potentially produce narrow NCs using low-cost scalable procedures. For instance, Liu et al.^23^ prepared NCs of two antitumoral drugs, paclitaxel and camptothecin, using Pluronic 127 as a stabilizer to increase the circulation lifetimes of both drugs. They achieved lower toxicity than the equivalent dose of the free drugs, and an excellent antitumor activity, besides, an easy scale-up manufacturing method was proposed. Antisolvent precipitation methods have been used to obtain NCs around 200 nm in size having different stabilizers, usually methylcellulose derivatives,^24,25^ poly(vinyl alcohol) (PVA),^26^ using various solvent/antisolvent combinations and also, in some cases, even without the use of any stabilizer.^27^ Stable NCs of mean particle sizes in the range of 10–20 nm were obtained using poly(ethylene glycol) (PEG300) as solvent for the dissolution of the drug (carbamazepine), and an aqueous solution of hydroxypropyl methylcellulose (HPMC) as antisolvent.^28^ The presence of the cellulose derivative inhibits crystal growth, and was found to decrease the particle size by its adsorption on the surface of hydrophobic drugs limiting the access for the dissolved drug in the medium to the growing crystallization nuclei. The high surface activity of methoxy and hydroxypropyl groups present in cellulose derivatives allows the interaction with drug molecules *via* hydrogen bonding, which inhibits crystal growth rendering crystals of a few nanometers in diameter, being nucleation promoted and crystal growth arrested. NCs have also been loaded within polymeric particles to promote their sustained drug release. For instance, Wang et al.^29^ developed poly(lactic-*co*-glycolic acid) (PLGA) microparticles (MPs) loaded with breviscapine (a naturally occurring flavonoid) NCs, using a water-soluble polymer template method, having an improved drug loading for a prolonged drug delivery representing a promising approach for long-term delivery of therapeutic doses.

Several nanomaterials have been proposed as drug delivery systems to obtain long-term analgesia in the management of chronic pain.^30^ A sustained-release system with long-term action is desired to satisfy the patients’ requirements, as well as to maintain constant therapeutic levels. Current available local anesthetics approved for single-injection with extended duration of action are, for instance, liposomal formulations of bupivacaine (Exparel) and morphine (DepoDur). Furthermore, on-demand triggerable nanoparticulated systems for achieving pulsatile anesthetic release profiles have also been reported. For instance, phototriggered local anesthesia was achieved using liposomes attached to gold nanorods as plasmonic nanoparticles that generate heat upon light stimulation to trigger the release of a local anesthetic drug (*i.e*., tetrodotoxin) on-demand.^31^

Herein, to achieve long-term local anesthesia, bupivacaine nanocrystals (BNCs) were prepared and encapsulated within biocompatible poly(ethylene glycol) methyl ether methacrylate (OEGMA)-based nanogels. To the best of our knowledge, this is the first time that BNCs are prepared and tested *in vivo* for pain management applications and benchmarked against the clinically used bupivacaine hydrochloride solution. One of the objectives of this study was to synthesize thermoresponsive biocompatible nanogels with high drug loadings. The ability of the nanogels loaded with bupivacaine nanocrystals (BNC-nanogels) to provide *in vivo* sciatic nerve blockade was successfully evaluated. Moreover, the potential *in vitro* and *in vivo* toxicity of the materials was assessed in this study.

## Results and Discussion

### Characterization of Nanogels

**Figure 1:**
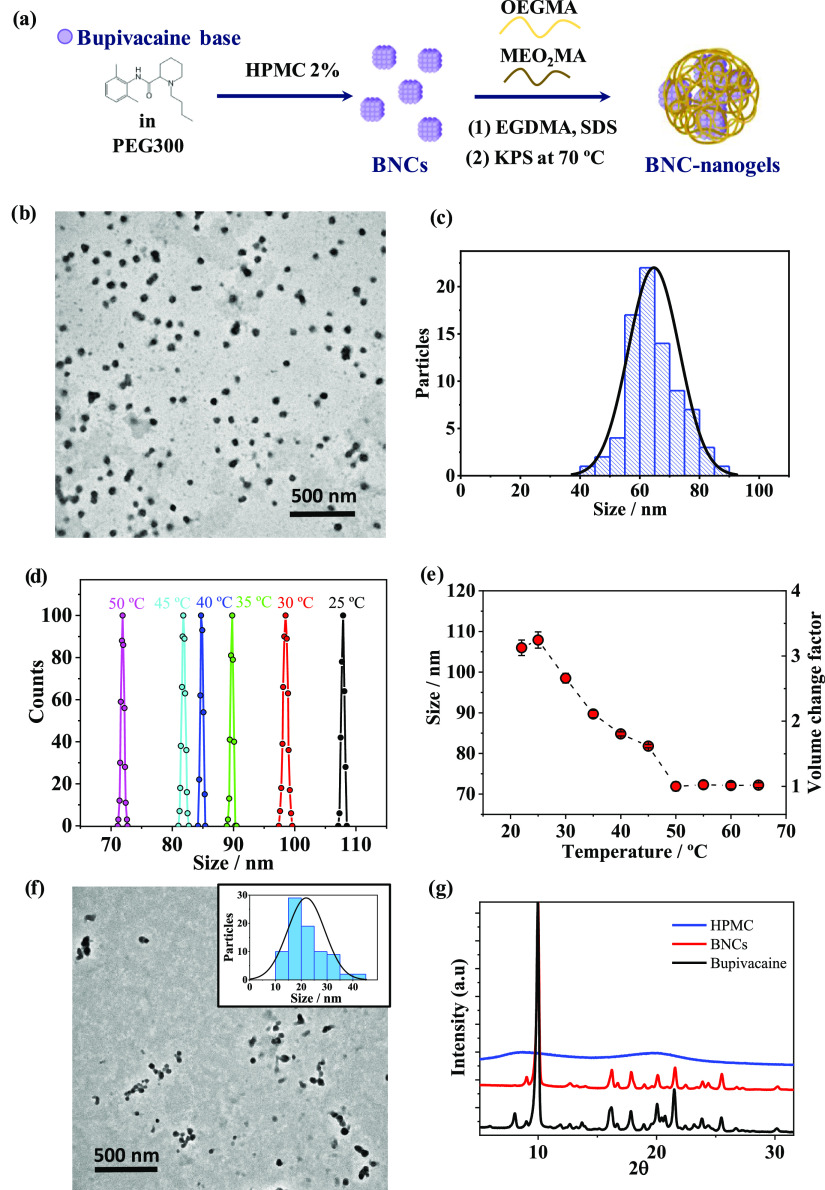
(a) Scheme of the global synthesis of BNC-nanogels. The first stage is the preparation of BNCs using antisolvent precipitation method. Second is the synthesis of thermoresponsive nanogels by *in situ* free-radical copolymerization of MEO_2_MA and OEGMA_500_ monomers in the presence of as-prepared BNCs. (b) Representative TEM image of thermoresponsive nanogels. (c) Size distribution histogram with Gaussian-fitting curve (solid line) for nanogels obtained from TEM images (*N* = 80). The average size was 64.7 ± 8.6 nm. (d) Size distribution dynamic light scattering (DLS) plots obtained for nanogels at different temperatures. (e) Variations in the hydrodynamic sizes and volume change factor of nanogels as a function of temperature measured using DLS. Data are mean ± standard deviation (SD) (*N* = 5). (f) TEM image presenting BNCs size and morphology; inset: size distribution histogram obtained from TEM images (*N* = 80). The average size was 21.9 ± 7.2 nm. (g) X-ray diffraction (XRD) diffractograms of bupivacaine, BNCs, and HPMC.

The preparation of thermoresponsive nanogels was performed in one-pot synthesis by *in situ* free-radical copolymerization of MEO_2_MA and OEGMA_500_ monomers, according to the procedure described in the Experimental Section,^32^ and as it is depicted in Figure 1a. Transmission electron microscopy (TEM) images of empty (*i.e*., drug-free) nanogels in Figure 1b show particles with spherical-like morphology of uniform size. The size distribution histogram with Gaussian-fitting curve (solid line) for nanogels shown in Figure 1c exhibits a narrow size distribution. The average size of the empty nanogels measured from TEM images (*N* = 80) was 64.7 ± 8.6 nm. The ζ potential of the nanogels dispersed in Milli-Q water was negative, with a value of −6.7 ± 0.9 mV. The negative surface charge is attributed to the sulfate groups present in the anionic surfactant used to stabilize the nanoparticles together with the steric hindrance of the OEGMA groups. Indeed, the nanogels obtained showed great stability, as they remained dispersed in solution during weeks without sedimentation and showing the same hydrodynamic size and ζ potential after at least 3 months of storage (results not shown). Also, their stability in water is the reason for the use of ultracentrifugation at 25 000 rpm to precipitate them and recover the nanogels from water.

The thermoresponsive properties of nanogels were evaluated using DLS. To accomplish this, the change in the hydrodynamic average size of nanogels dispersed in Milli-Q water was determined at different temperatures from 22 to 65 °C. Before each DLS measurement, the temperature was equilibrated in the sample holder at least for 5 min. The hydrodynamic size distributions shown in Figure 1d are highly monodisperse, with polydispersity index (PDI) values between 0.001 and 0.09. Temperature-induced shrinking of nanogels was observed in the plot. Hydrodynamic nanoparticle sizes are consistent with TEM results, as the larger size obtained by DLS is explained by the effective swelling of the nanogels in aqueous dispersions. Conversely, the smaller size obtained by TEM is the one of the dry dehydrated shrunken nanogels under the high-vacuum conditions used.

The thermoresponsive behavior of the obtained nanogels is presented in Figure 1e with the plot of hydrodynamic size versus temperature, where the reduction in the size of nanogels with the increase in temperature is due to the collapse of the nanogels above their volume phase transition (VPT). The inflection point of the hydrodynamic size versus temperature curve is defined as the volume phase transition temperature (VPTT) of the nanogels. The nanogels with VPTT above the physiological temperature (37 °C) are suitable as drug carriers. The VPTT obtained was 38.3 °C, which is in accordance with our previous studies on lower critical solution temperature (LCST) of the equivalent OEGMA-based copolymers.^33^ The produced nanogels were able to reduce their size from 107.9 nm at 25 °C (swollen) to 72.1 nm at 60 °C (shrunken), giving a volume change factor of 3.3. Therefore, they undergo 70% reduction in their volume and hydrophilic/hydrophobic character, which allows them to be used as drug vehicles to release their cargo upon temperature changes in a reversible manner. Furthermore, rheological analysis was performed to study the phase transition of the nanogels (Figure S1). VPTT can be determined through tracking the evolution of storage modulus (*G*′) and loss modulus (*G*″) in dynamic temperature sweep tests. Results in Figure S1 show that below the transition temperature *G*′ and *G*″ remained constant with temperature; however, when the phase transition was reached, the enhancement of the system elasticity resulted in a sharp increase in *G*′ and *G*″ moduli. The critical temperature at which *G*′ and *G*″ rapidly increase (40.5 °C) is considered the VPTT. When the temperature increases until reaching the VPTT, intermolecular and intramolecular attractive interactions between polymer chains increase and dehydration occurs, which causes an increase in the rigidity of nanogels and therefore an abrupt increase in *G*′ and *G*″.^34^ The slight difference in the VPTT obtained by DLS and by rheological analysis can be ascribed to the difference in the analytical technique used, since in DLS, we determined the volume change of nanogels as colloidal suspensions, and in rheological tests, we analyzed the changes in their viscoelastic properties. Nevertheless, both methods displayed consistent results, giving a close value for VPTT.

### Characterization of BNC-Nanogels

With the aim to obtain high drug loading contents, the local anesthetic bupivacaine was nanocrystallized. The NCs were prepared from the hydrophobic form of bupivacaine (bupivacaine free base) using the antisolvent precipitation technique, before being introduced in the precursor solution for the synthesis of nanogels. As far as we know, this is the first time that BNCs are reported. TEM studies were performed to examine the size distribution and morphology of the obtained BNCs. TEM images showed spherical NCs of 21.9 ± 7.2 nm in size with a narrow size distribution (Figure 1f). The XRD technique was used to characterize the BNCs crystallinity and the size of their elementary crystallites (*i.e*., unit cell dimension). The most representative XRD diffraction planes of bupivacaine reference (Figure 1g) were located at 10, 16.2, 17.8, 20, 21.5, 23.8, and 25.5°. The spectrum of the obtained BNCs matched most of the reference diffraction planes, indicating that the BNC structure remains in a well-crystallized state. The XRD spectra of HPMC used as a stabilizer agent and empty nanogels showed the characteristic plots of amorphous compounds. The mean size of the BNCs elementary crystallites can be determined using Scherrer’s equation.^35^ Analyzing the five most intense peaks of the spectra, the size of BNCs obtained using the Scherrer constant for spherical crystallites (*K* = 0.94) using the Voigt fitting model was 35.93 ± 1.1 nm. This value is in good agreement with the microscopy characterization. The drug loading obtained by GC-MS for BNCs was 93.8 ± 1.5 wt %. BNCs are therefore mainly consisting of drug crystals having a small fraction of HPMC.

To obtain BNC-nanogels of high drug content, the BNCs were introduced during the synthesis of the nanogels, with the purpose of growing the nanogels on the surface of the crystals. The encapsulation of NCs could be mainly driven by hydrophobic interactions of highly methylated glucose zones in HPMC^36^ with the main polymer backbone having hydrophobic character, keeping the drug in the core of the nanoparticles. The overall scheme representing the synthesis of BNC-nanogels is depicted in Figure 1a.

**Figure 2:**
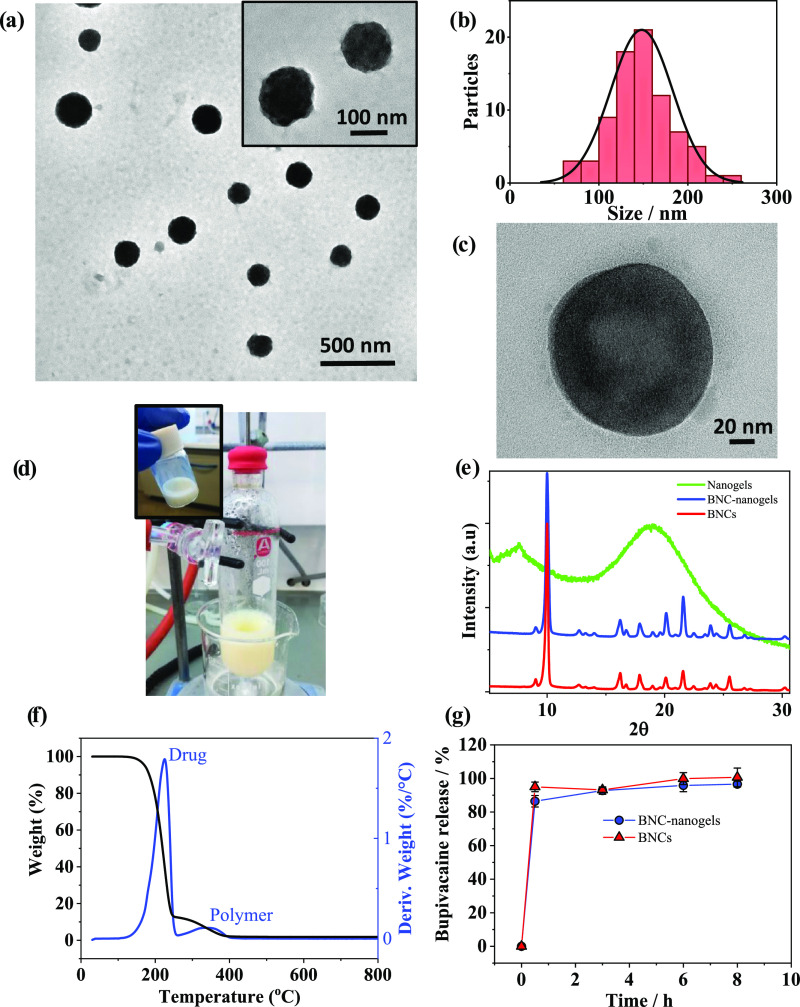
Electron microscopy analysis of BNC-nanogels: (a) representative TEM images of BNC-nanogels. (b) Size distribution histogram obtained from TEM images with a number of elements *N* = 80. The average size was 148 ± 35 nm. (c) High-resolution TEM image showing in detail a nanogel embedding BNC. (d) Photograph of BNC-nanogels dispersed in aqueous solution. (e) XRD patterns recorded for BNCs, nanogels loaded with BNCs and empty nanogels. (f) TGA (black) and derivative of TGA (blue) plots for BNC-nanogels. (g) Bupivacaine release profiles from BNCs (nonencapsulated) and BNC-nanogels at 37 °C. Data are mean ± SD (*N* = 3).

Figure 2a,b shows TEM images and size distribution histogram of BNC-nanogels, respectively. It can be seen that nanogels presented a uniform size with a high electronic density in the core due to the BNCs embedded in their interior (Figure 2a,c). The size of the BNC-nanogels was 148 ± 35 nm. This size is twice the one of empty nanogels presented in Figure 1b. The considerably larger size of the BNC-nanogels indicates the encapsulation of the drug crystals inside. Photographs of the obtained BNC-nanogels dispersed in aqueous solution are shown in Figure 2d. The XRD characterization of BNC-nanogels in Figure 2e shows the characteristic bupivacaine diffraction planes, which demonstrates that nanocrystals were present in the nanogels. The ζ potential of the BNC-nanogels remains in the same range as the one for empty nanogels, with a value of −6.91 ± 0.38 mV. BNC-nanogels were analyzed by thermogravimetric analysis (TGA) in the temperature range of 30–800 °C. Figure 2f shows the weight loss of the nanogels embedding BNCs as a function of temperature. The thermal decomposition of the BNC-nanogels occurred between 150 and 400 °C. At 400 °C, the weight loss corresponds to the total initial mass. Bupivacaine hydrochloride total thermal decomposition takes place at 266 °C (see Figure S2), and the polymer P(MEO_2_MA-*co*-OEGMA_500_) decomposition occurs between 250 and 400 °C.^37^ The derivative of the weight loss (Figure 2f) presented two defined peaks centered at 260 °C, attributed to the bupivacaine thermal decomposition, and at 340 °C, compatible with the polymer loss (Figure S2). Therefore, the polymer content in the final formulation and drug content were estimated to be 13 wt % and 87 wt %, respectively. The partial overlap of the pure polymer and drug curves does not allow us to precisely determine the mass of each component by this technique; however, the estimated value was consistent with the DL% obtained using gas chromatography–mass spectrometry (GC-MS).

The quantitative determination of the drug incorporated into nanogels and its posterior *in vitro* release were performed by GC-MS. The drug loading of BNC-nanogels determined by GC-MS was calculated as 84.8 ± 1.2 wt %. To the best of our knowledge, this is one of the highest bupivacaine loadings reported in polymeric nanoparticles, even superior to the results obtained by Curley et al.^2^ who obtained PLGA microspheres loaded with 75 wt % bupivacaine free base using oil-in-water (o/w) emulsification and solvent evaporation method. The drug release profiles of BNC-nanogels and BNCs are shown in Figure 2g. The dispersions were prepared to have the same drug amount in both samples according to the drug loading (0.196 mg for BNC-nanogels and 0.180 mg for BNCs in 1 mL aqueous solution) and to work below the drug saturation concentration of the bupivacaine free base form (0.4 mg/mL) as working under sink conditions.^38^ Results showed a fast release with an onset reached in less than 30 min when the drug concentration was below the saturation concentration. As it is shown in Figure 2g, the release of BNCs and BNC-nanogels is quite similar and the dissolution of the drug occurs in the first hours under conditions below the drug saturation concentration. The dissolution rate of nanocrystals composed of the bupivacaine free base will be limited by the low aqueous solubility of this nonionized form (0.4 mg/mL), in contrast to bupivacaine hydrochloride salt having a solubility 10-fold higher (20–40 mg/mL).^38^ To analyze the kinetics of drug release from BNCs and BNC-nanogels, release results were fitted to different mathematical models: zero order, first order, Higuchi, Korsmeyer–Peppas and Hixson–Crowell. The best model was selected according to the correlation coefficient (*R*^2^) determined from the linear regression fit for each model. The obtained kinetic data for the drug release together with the correlation coefficients (*R*^2^) are listed in Table S1. According to the regression results, the release profile of BNC-nanogels (*R*^2^ = 0.998) and BNCs (*R*^2^ = 0.999) followed the Korsmeyer–Peppas model with kinetic constant *K*_BNC-nanogels_ = 0.884 h^–*n*^ and *K*_BNCs_ = 0.963 h^–*n*^. The value of the kinetic constant decreased when BNCs were encapsulated in the nanogels due to the increased viscosity and governance of polymeric chain entanglement.^39^ The parameters obtained for the release exponent (*n*_BNC-nanogels_ = 0.041 and *n*_BNCs_ = 0.021) less than 0.5 indicate that the drug release was governed by Fickian diffusion as reported by other authors for thermoresponsive polymeric nanoparticles.^40^ The initial rapid drug release has been attributed to the fast dissolution rate of small NCs^41^ and the presence of drug molecules that are located close to the external surface, but embedded in the polymeric matrix.^42^

### *In Vitro* Biological Assays

The *in vitro* cytotoxic effects of the synthesized materials were studied in four different cell lines: human dermal fibroblasts, macrophages, mouse mesenchymal stem cells (mMSCs), and U251MG. After treatment for 24 h, the effects on cell metabolism, apoptosis, and cell cycle were assessed. Moreover, the endotoxin levels of BNCs and BNC-nanogels were evaluated to ensure their safety for their *in vivo* application.

**Figure 3:**
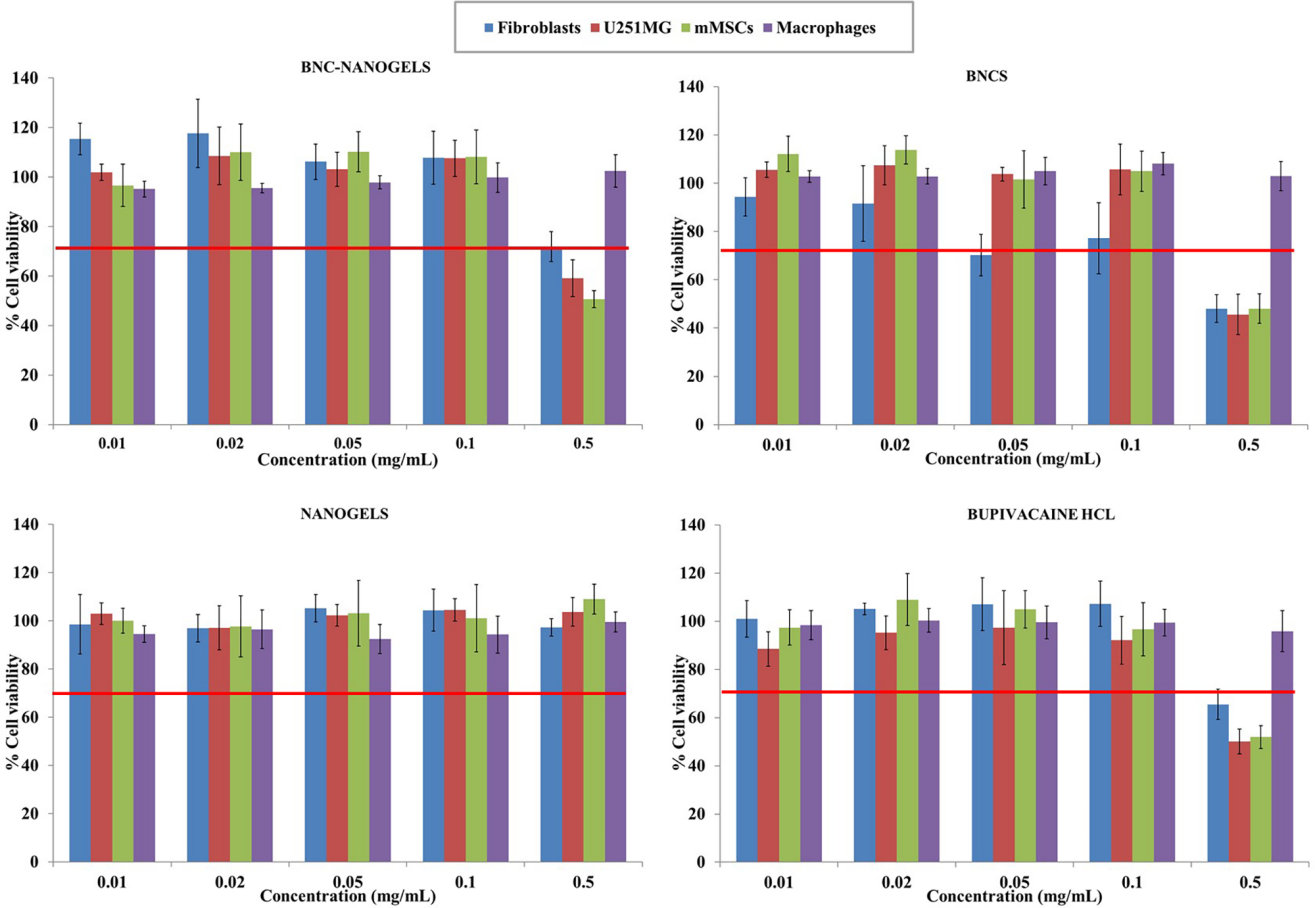
Cell viability of BNC-nanogels, BNCs, empty drug-free nanogels, and bupivacaine hydrochloride in the four cell lines assayed after 24 h. The red line depicts the threshold of 70% of cell viability in accordance with ISO 10993-5. Percentages are displayed as mean ± SD (*N* = 5).

The treatment of the cell lines described above for 24 h with BNC-nanogels, BNCs, empty drug-free nanogels, and bupivacaine hydrochloride solution produced the effects depicted in Figure 3. The assays were performed at BNC-nanogel concentrations ranging from 0.01 to 0.5 mg/mL and at the equivalent drug concentrations, for BNCs and free bupivacaine hydrochloride, or the equivalent polymer concentration in the case of empty nanogels. Empty thermoresponsive nanogels did not reveal cytotoxic effects as cell viability was found in all concentrations and cell lines tested above 92% in compliance with ISO 10993-5,^43^ which describes that a reduction in cell viability higher than 30% compared to the control sample is considered as cytotoxic. These results are in accordance with previous results of our group in which the thermoresponsive polymer P(MEO_2_MA-*co*-OEGMA_500_) synthesized by photopolymerization exerted cell viability percentages higher than 92% at concentrations up to 0.4 mg/mL.^33^ On the other hand, BNCs and bupivacaine hydrochloride involved a decrease in cell viability (∼50%) at the highest concentration assayed (0.5 mg/mL) except for THP1-derived macrophages, which maintained cell viability in the same range as the control sample. Finally, BNC-nanogels also showed some cytotoxic effects in U251MG and mMSCs at the highest concentration studied displaying viabilities around 59 and 50%, respectively. However, percentages higher than 70% were recorded in fibroblast and macrophage samples at all of the concentrations assayed. It should be noted that at the highest concentration assayed, BNC-nanogels displayed higher viability percentages than BNCs pointing to the potential protective effect of the nanogel by including inside its structure the BNCs, except for macrophages whose percentages were in the range of the control sample (100%). With these results, the subcytotoxic dose for further experiments for BNC-nanogels, BNCs, and bupivacaine hydrochloride was considered 0.1 mg/mL, whereas for the empty nanogels, 0.5 mg/mL was selected. Previous works in our group have revealed similar results regarding the treatment of these cell lines with bupivacaine loaded in hollow gold nanoparticles (HGNPs) functionalized with the thermoresponsive polymer disulfide-P(MEO_2_MA-*co*-OEGMA_500_),^37^ bupivacaine loaded in a cleavable nanocomposite composed of CuS nanoparticles and POEGMA (CuS-P(MEO_2_MA-*co*-OEGMA_500_)),^44^ and in hybrid poly(*N*-isopropylacrylamide) (PNIPAm)-based nanogels decorated with plasmonic hollow gold nanoparticles (HGNPs-PNIPAm) loading bupivacaine.^45^ At 0.1 mg/mL, these prior works showed cell viability percentages lower than those displayed in the present work, probably owing to the presence of free bupivacaine, HGNPs, or CuS nanoparticles, except for macrophages^37^ and U251MG.^45^

Cell membrane damage was assessed by means of cell apoptosis studies developed by flow cytometry (Figures S3 and S5). The results obtained did not reveal remarkable differences between control and treated samples. Only BNCs treatment in fibroblasts and macrophages involved a slight increase (≤10%) in total apoptosis rate (early apoptosis + late apoptosis) and necrosis percentage, respectively. Furthermore, U251MG recorded an increase in the total apoptosis rate of 12–16% in regard to the control sample when bupivacaine hydrochloride or BNCs were present in the samples. These results are in accordance with our previous results related to cell apoptosis after treatment for 24 h with PNIPAm-based nanogels loaded with bupivacaine,^45^ where free bupivacaine treatment also involved a change in apoptosis, necrosis, and viability percentages in U251MG cells.

The distribution of cell cycle phases was evaluated by flow cytometry (Figures S4 and S5). Cell treatment with BNC-nanogels, BNCs, empty drug-free nanogels, and bupivacaine hydrochloride solution for 24 h did not exert significant changes in cell cycle, displaying changes in percentages up to 10% which is in agreement with previously reported results for bupivacaine-loaded HGNPs-PNIPAm nanogels.^45^ However, a superior effect was observed in macrophages whose S phase was increased (3–23%) with a countervailing reduction in the G1 and G2 phases when cells were treated with the materials assayed showing a higher effect when nanogels were present in the sample. Therefore, cell treatment with these materials was not detrimental for DNA and cell nuclei as cell cycle was not significantly affected.

Finally, endotoxin levels in BNCs and BNC-nanogels were quantified by means of the Pierce Chromogenic Endotoxin Quant Kit as mentioned before. The endotoxin concentrations obtained from these samples were much lower than the approximate threshold pyrogen dose for humans (0.05 ng/mL = 0.5 endotoxin unit (EU)/mL) for a nonintrathecal administration, as indicated by the FDA.^46^ In conclusion, the synthesized thermoresponsive nanogels loaded with BNCs did not significantly affect cell metabolism, membrane, and cycle on the cell types studied, as well as it did not involve potential detrimental endotoxin levels in a potential *in vivo* administration, pointing to their promising biomedical application in peripheral nerve pain relief.

### *In Vivo* Sciatic Nerve Blockade

**Figure 4:**
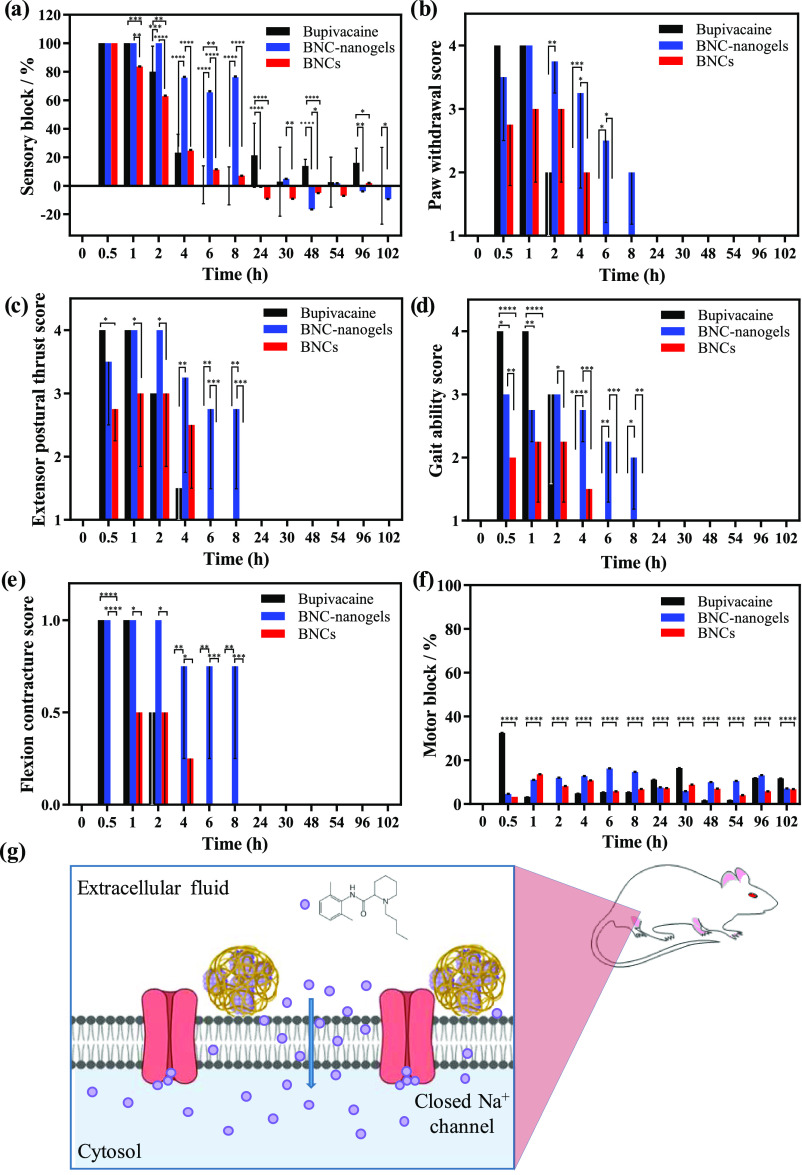
Sciatic nerve blockade effect for rats injected with free bupivacaine hydrochloride, nanogels embedding BNCs and BNC formulations. (a) Sensory nerve blockade using the hot plate test. Behavioral reflexes: (b) paw withdrawal test scores and (c) adapted extensor postural thrust scores. (d) Gait ability test scores. (e) Flexion contracture test scores. (f) Motor nerve blockade using weight bearing test. (g) Scheme showing the *in vivo* mechanism proposed for the nerve blockade using BNC-nanogel formulations. Score 1 represented a normal reflex or response, while score 4 denoted the absence of reflex or response. The nonexistence of contracture was assessed as 0 and as 1 if the foot showed a flexion contracture. Data are presented as mean ± SD (*N* = 4 per group) (*p < 0.05, **p < 0.01, ***p < 0.001, and ****p < 0.0001).

Rats were injected close to the sciatic nerve root with the drug formulations containing all of the same bupivacaine equivalent dose of 2 mg. The injected dispersions were: 43 mg/mL BNCs (94% bupivacaine loading), 47 mg/mL BNC-nanogels (85% bupivacaine loading), and 40 mg/mL bupivacaine hydrochloride solution. The sensory nerve blockade was evaluated using the hot plate test. Results in Figure 4a showed that all of the bupivacaine-containing injected formulations, BNC-nanogels, BNCs, and free bupivacaine hydrochloride, achieved the maximum sensory nerve blockade (thermal latency higher than 8 s) by the time of the first measurement, that is, 30 min after injection. The nerve blockade remained 80% after 2 h for the free bupivacaine hydrochloride administration, and the normal sensory nerve behavior was recovered between 4 and 6 h after treatment. In the case of BNCs, the sensory nerve blockade gradually decreased in the first 6 h reaching at that time the 10% of the maximum effect. However, results in Figure 4a indicated that BNC-nanogels produced a longer duration of sensory nerve blockade in comparison to the effect of the nonencapsulated BNCs and the one of the free bupivacaine hydrochloride solution. The sensory nerve blockade for BNC-nanogels was effective for at least 8 h after injection, remaining at this time the 76% of the maximum nerve block. This outcome suggested that the OEGMA-based nanogels were able to retain the bupivacaine in the proximity of the sciatic nerve prolonging the anesthetic effect more than 8 h with the same dose of drug (2 mg) as the ones contained in the other formulations. Probably, this effect is attributed to the thermoresponsive character of the polymer, which, when reaching physiological temperature, becomes hydrophobic, shrinks, and packs the nanocrystals encapsulated in its interior acting as a diffusion barrier for the drug remaining isolated from the medium due to its hydrophobic nature and producing a sustained release. Obviously, there is no correlation between the *in vitro* (Figure 2g) and *in vivo* effect observed; therefore, the interaction of the material with the tissues plays a very important role in the prolonged duration of action observed.

The evaluation of the two behavioral reflexes or responses pointed out the same trend as the sensory nerve block test. For the free bupivacaine hydrochloride formulation, the paw withdrawal tests showed a score of 4 (Figure 4b), with total absence of mechanical sensitivity for 1 h after injection. Also, for this formulation, the adapted extensor postural thrust analysis showed no flexor function in the animals, the presence of a flexor contracture in the treated hind paw, along with a total lack of mobility in the limb during the first hour (Figure 4c–e). Two hours after the administration of the free bupivacaine hydrochloride, the animals had almost recovered the paw withdrawal reflex, the flexor contracture in the treated hind paw was reduced, showing a half-open paw and they had recovered partially the movement of the leg. The only parameter that remained affected up to 4 h after administration was the flexor function that was totally recovered after 6 h of study. The BNCs showed anesthetic efficiency that lasted until 4 h of study (Figure 4a); however, differences can be observed in the behavioral responses and in the scores analyzed compared to the effect of the free bupivacaine hydrochloride solution. Animals treated with the BNCs showed a delayed mechanical sensitivity reflex and a weak grip (scores between 2 and 3 in Figure 4b,c), but the reflexes were not totally impeded. The animals showed a slight hindered movement of the limb (scores between 1.5 and 2.3 in Figure 4d) and a slight flexor contracture in the treated hind paw (Figure 4e). Therefore, the functionality of the limb was not totally lost as in the case of bupivacaine hydrochloride solution. Finally, the effect of administrating BNC-nanogels was different; they exhibited a more prolonged anesthetic effect with an extended duration of action persisting for more than 8 h (Figure 4a). Again, the motor function of the leg was not completely hindered, as the behavioral responses showed a slight response to paw withdrawal and the adapted extensor postural thrust analyses (Figure 4b,c). Also, the movement was slightly hindered (scores between 2 and 3 in Figure 4d) and a flexor contracture in the treated foot was somehow detected during 8 h (Figure 4e). These findings confirmed the prolonged effectiveness produced by the encapsulation of BNCs into nanogels. Saline controls were also performed, and no anesthetic effect was found (results not shown). Motor nerve blockade was analyzed for the different formulations using the weight bearing test. Results in Figure 4f showed a slight influence on motor block for BNCs and BNC-nanogel formulations that is compatible with the preservation of some reflexes in the limb, with exception of bupivacaine hydrochloride solution during the first hour of administration, where there was a superior motor nerve blockade.

Previous investigations using bupivacaine-loaded PLGA microparticles to produce sciatic nerve blockade^47^ did not achieve the maximal nerve block by the first 30 min although they administrated 38.5 mg of bupivacaine hydrochloride. This initial onset might be attributed to the slow degradation of PLGA by hydrolysis of the ester bond present in the backbone, facilitating a controlled release of encapsulated cargoes. These PLGA microparticles achieved a duration of sensory block of more than 11 h, providing a prolonged duration of the local anesthesia. Other authors^2^ reported a nerve block duration of 6 h using PLGA microparticles loaded with a total dose of 50 mg of bupivacaine hydrochloride. Other formulations reported in the literature include bupivacaine-loaded liposomes,^48^ which reached a duration of sciatic nerve block of 7.3 h, upon administration of liposomes containing 6 mg of bupivacaine. Our formulation achieved a continuous extended nerve blockade, for more than 8 h, with a total dose of only 2 mg of nanocrystallized bupivacaine. The hydrophobicity of bupivacaine free base probably facilitates its pass through cell membranes and also binds strongly to the hydrophobic lipid bilayer, thereby protecting molecules from diffusion to extracellular media and to the bloodstream, reducing their clearance in comparison to the ionized form (hydrochloride salt).^49,50^ Probably bupivacaine being a weak base diffused in its unionized form from the BNC-nanogel formulations to the interior of the cell, which ionized due to the slight acidic interior environment blocking the inner surface of the voltage-gated sodium channels avoiding action potential to propagate. Moreover, this extended duration of blockage is probably also explained by the immobilization of BNC-nanogels in the injection site around the sciatic nerve due to their hydrophobic character, preventing particle diffusion and fast drug clearance. At 37 °C, the nanogels are partially collapsed to half of their volume (Figure 1g), giving a transition to a more hydrophobic structure reducing their elimination from the body, interacting efficiently with the tissues and with the afferent nerve fibers. The hydrophobic character of the partially collapsed nanogel could retain the formulation in the injection site maintaining drug levels in the therapeutic window, which explains the different effect between nonencapsulated and encapsulated BNCs. A schematic illustration of the mechanism proposed for the regional anesthesia effect obtained for BNC-nanogels is depicted in Figure 4g. Other authors reported the immobilization of PLGA microparticles (MPs) embedded in thermosensitive macrogels based on PLGA–PEG–PLGA to obtain high local drug concentration for long-acting analgesia.^51^ Those PLGA MPs were prepared using oil-in-water (o/w) emulsification method for the encapsulation of bupivacaine free base. PLGA MPs containing 40 mg of bupivacaine were administrated embedded in a PLGA–PEG–PLGA solution having a sol–gel transition when exposed at body temperature (37 °C). They achieved a 22 h block of sciatic nerve, which is more than twice the duration accomplish by our BNC-nanogels, but using 20 times more amount of drug. In our formulation, we used nanocrystallized bupivacaine, which produced sustained release and enhanced the long-term nerve block.

In general, for several applications, it is desirable to obtain a longer duration of sensory block than motor block, for instance, in obstetric anesthesia where the mother should maintain the motor functionality while still obtaining pain relief. In the case of peripheral nerve pain treatment, something similar occurs; it is desirable for sensory block to be of longer duration than motor block to relieve the pain but not resulting in a paralyzed limb. Using BNC-nanogels, we have obtained still mobility in the limb causing sensory nerve block enough to relieve the pain. The amount of myelin around the nerve axons is different in afferent (*i.e*., nociceptive sensory fibers innervated in the direction toward the bone marrow) than that in efferent (*i.e*., motor neurons that exit the bone marrow innervating the skeletal muscles) nerve fibers; therefore, a specific anesthetic dose is able to block the nerve impulse transmission in one direction (sensory block) while allowing the motor neurons to carry efferent impulses to the effector in the other direction, which results in movement. Another important aspect to take into account is the lack of observed *in vivo* toxicity exhibited after the treatment with BNC-nanogels. Other polymeric bupivacaine-loaded delivery systems have reported local toxicity, myotoxicity, and inflammation probably attributed to a fast release kinetics of bupivacaine (burst release), and especially to the accumulation of polymeric residues that remain attached to the tissues.^52−54^ The *in vivo* sustained-release kinetics of the BNC-nanogels among with the excellent biocompatibility of PEG-based nanogels are the reasons for the low toxicity exhibited by these nanomaterials. Therefore, BNC-nanogels can be potentially used in postoperative pain control by local infiltration, for instance, in mammoplasty, total knee arthroplasty, hemorrhoidectomy, and inguinal hernia repair. BNC-nanogels are excellent candidates for epidural uses as they allow motor functionality while obtaining pain relief. In this sense, they could be used in peripheral nerve blockage as a promising treatment for the management of sciatica, which is the most common cause of chronic neuropathic pain.

### Tissue Reaction

**Figure 5:**
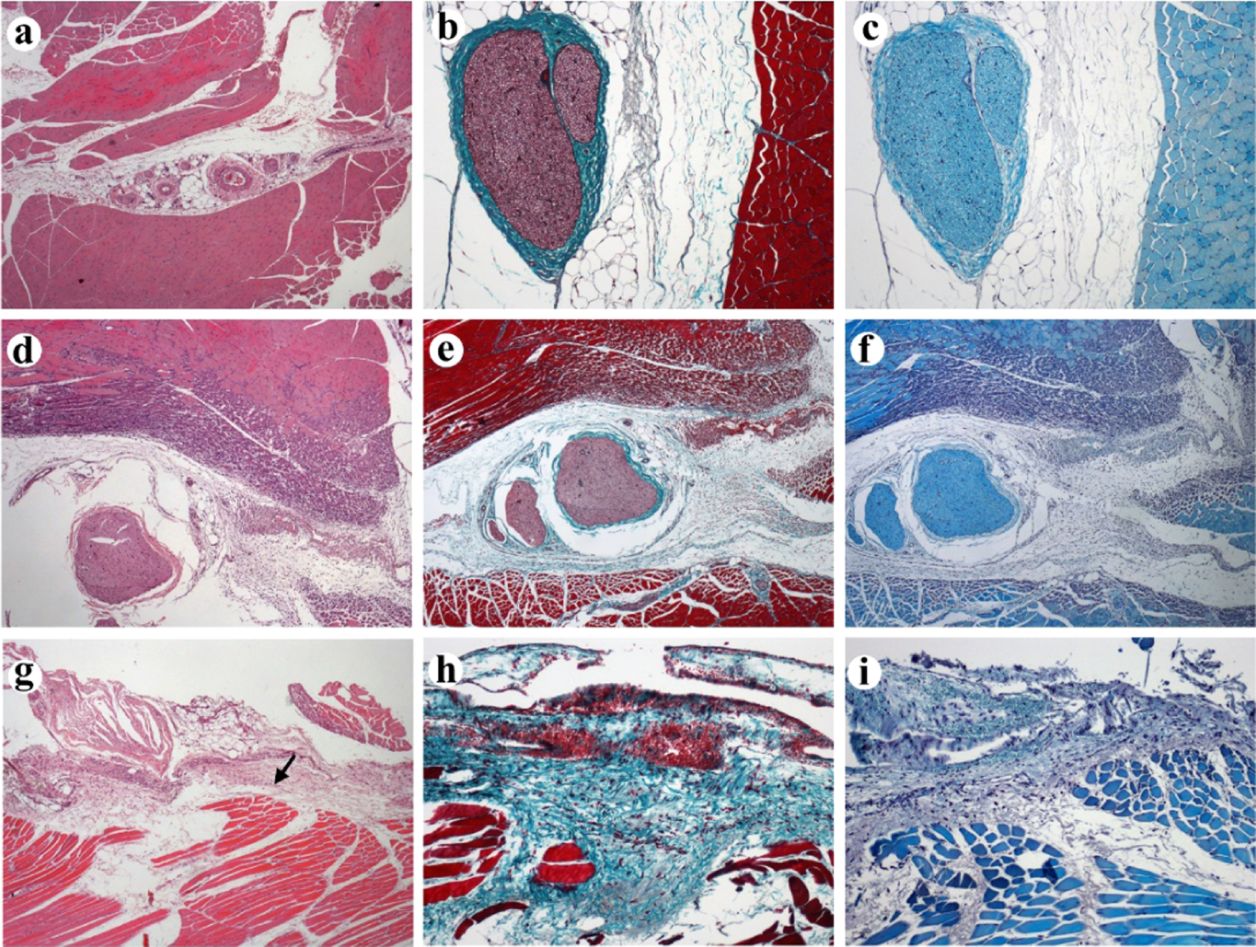
Tissue sections of the sciatic nerve root area collected 4 days post-inoculation (dpi): (a–c) Rat injected with bupivacaine nanocrystals (BNCs) nanogels. No significant changes in nerve, muscular, and adipose tissues are observed. (a) Hematoxylin–eosin (HE) 4×, (b) Masson′s trichrome 10×, and (c) Luxol fast blue 10×; (d–f) rat injected with BNCs. Mild to moderate lesions around the injection site including fibroplasia and muscular fiber regeneration. (d) HE 4×, (e) Masson’s trichrome 4×, and (f) Luxol fast blue 4×; (g–i) rat injected with free bupivacaine hydrochloride. No significant changes are observed. There is a mild fibrous reaction along the injection site (arrow). (g) HE 4×, (h) Masson’s trichrome 10×, and (i) Luxol fast blue 10×.

Sciatic nerve and surrounding tissues were collected 4 days after administration, and formalin-fixed, paraffin-embedded sections were stained with HE, Masson, and Luxol Fast Blue staining to evaluate the response of the connective, adipose, and nervous tissues against the inoculated substances. Our findings (Figure 5) suggest that BNC-nanogel administration elicited minimal tissue reaction with no discernible influence on the sciatic nerve (Figure 5a–c). However, the BNC group (Figure 5d–f) showed a mild to moderate tissue reaction around the injection site, including moderate fibroblastic proliferation and mild muscle fiber regeneration. These findings highlight a minimal damage of the polymer for the tissue. On the other hand, the free bupivacaine group (Figure 5g–i) did not show significant tissue lesions apart from mild fibroblastic proliferation and edema. These promising results support the potential clinical use of BNC-nanogels regarding their effectiveness in sciatic nerve blockage and their biocompatibility in the tissues.

Overall, the incorporation of BNCs into nanogels has shown superior drug loading compared to other reported polymeric nanocarriers.^2^ In comparison to other authors reporting the encapsulation of bupivacaine in thermosensitive systems, at an equivalent dose administered, our vector achieves at least 3 times longer duration of action in sciatic nerve block.^2,47,48,51^ In addition, the advantage of BNC-nanogels is that they produce a sensory block that lasts longer than motor block, which still allows mobility in the limb but causes sufficient sensory nerve block to relieve pain. The histology results showed a lack of *in vivo* toxicity in contrast to the reported toxicity observed for other delivery systems based on bupivacaine.^52−55^ Exparel, a commercially available multivesicular liposomal formulation of encapsulated bupivacaine, has been tested *in vivo* for sciatic nerve blockade in rats^56^ following a similar output to the one reported in this work. Exparel injection containing 7.8 mg of bupivacaine hydrochloride caused a sciatic nerve blockade in rats lasting 4 h compared to more than 8 h for our BNC-nanogels containing only 2 mg of bupivacaine free base. Moreover, the previously reported histological study 4 days after the injection showed that inflammation scores for Exparel group were higher than those reached in animals treated with the same dose of bupivacaine solutions. In contrast, myotoxicity scores in animals treated with Exparel were not significantly different from the group containing the same dose of free bupivacaine solution. In our case, the outstanding biocompatibility of BNC-nanogels resulted in low cytotoxicity and no inflammatory response according to the *in vivo* studies.

## Conclusions

In this work, thermoresponsive nanogels loaded with BNCs were developed having a high drug loading content for a prolonged duration of regional anesthesia. BNCs were prepared from bupivacaine free base using the antisolvent precipitation method and subsequently encapsulated in poly(ethylene glycol) methyl ether methacrylate (OEGMA)-based nanogels by in situ free-radical copolymerization. The resulted BNC-nanogels were monodisperse in size and rendered a drug content of 84.8 ± 1.2 wt. %. The nanogels presented a VPTT above the physiological temperature at which they suffer a reduction of 70% in their volume, becoming a more hydrophobic structure and affecting the release of the contained cargo. The *in vivo* administration of BNC-nanogels containing 2 mg of drug achieved more than 8 h of sciatic nerve block in rats, whereas the same amount of free bupivacaine solution produced only 2–4 h of anesthetic effect. The hydrophobic character of the bupivacaine free base present in the nanogels probably facilitates its pass through cell membranes and also binds strongly to the hydrophobic cellular lipid bilayer, thereby protecting molecules from diffusion to extracellular media and to the bloodstream, reducing their clearance in comparison to its ionized form. Moreover, this extended duration of action can also be explained by the regional immobilization of the BNC-nanogels in the injection site around the sciatic nerve due to their hydrophobic character, preventing particle diffusion and fast drug clearance while interacting efficiently with the tissues and with the afferent nerve fibers. In addition, the BNC-nanogels presented low cytotoxicity and no inflammatory response in the *in vivo* administration in murine models due to the sustained drug release and the excellent biocompatibility of the OEGMA-based nanogels. The encapsulation of drug nanocrystals is a promising strategy for prolonged local anesthesia, reducing the total amount of drug necessary to produce pain relief with the consequent benefits obtained from their reduced associated toxicity.

## Experimental Section

### Materials and Methods

Di(ethylene glycol) methyl ether methacrylate (MEO_2_MA, 95%), poly(ethylene glycol) methyl ether methacrylate (OEGMA_500_, *M*_n_ 500), ethylene glycol dimethacrylate (EGDMA, >98%), sodium dodecyl sulfate (SDS, ≥98%), potassium persulfate (KPS, ≥99%), hydroxypropyl methylcellulose (HPMC, *M*_n_ ∼10 000), poly(ethylene glycol) (PEG300, *M*_n_ 300), bupivacaine hydrochloride monohydrate (≥99%), and (*S*)-(−)-limonene (96%) were purchased from Sigma-Aldrich. All of these chemicals were used as received.

The drug content of the formulations was determined using a gas chromatograph–mass spectrometer (GC-MS QP2010 SE, Shimadzu, Kyoto, Japan) equipped with an AOC-20i autoinjector. The mass spectrometer was set at an interface temperature of 280 °C and an ion source temperature of 200 °C, with a mass range of 35–300 *m*/*z* and a solvent cut time of 2.5 min. The capillary column used to separate the compounds was a Zebron ZB-50 (Phenomenex) having dimensions 30 m (length) × 0.25 mm (diameter) × 0.25 μm (film thickness). The injector temperature was adjusted at 250 °C, and the split mode was used at a 10:1 ratio. The GC oven temperature was initially set at 50 °C and further increased to 170 °C using a ramp rate of 30 °C/min, then raised at 45 °C/min to 250 °C (0.5 min hold), and finally raised at 10 °C/min to 300 °C and held there for 1.5 min. The carrier gas used was helium (>99.999%) with a flow linear velocity of 31 cm/s. The system was programmed with 11 mL/min of total flow rate and 0.73 mL/min of column flow. Bupivacaine was identified by its retention time (RT) and mass fragmentation pattern. Bupivacaine was quantified by the peak area comparison relative to the one of limonene used as an internal standard. Data peak processing was carried out by means of Shimadzu GC-MS solution software.

The hydrodynamic size of the nanogels and their volume phase transition *vs* temperature were measured using dynamic light scattering (DLS) on a Brookhaven 90Plus particle size analyzer with a detection angle of 90°. Measurements were made at different temperatures ranging from 20 to 65 ± 0.1 °C. The ζ potential of the nanogel-based colloidal suspensions was measured on a Brookhaven 90Plus particle size analyzer using ZetaPALS software in 1 mM KCl aqueous solution at a pH = 6 and 25 ± 0.1 °C. The ζ potential was determined by studying their electrophoretic mobility and then applying the Smoluchowski equation. Thermogravimetric analysis (TGA; Mettler Toledo TGA/STDA 851e) was carried out in a temperature range between 30 and 800 °C using a heating rate of 10 °C/min, under nitrogen atmosphere with a flow rate of 50 mL/min.

The nanogels were also characterized by rheological assays using a stress-controlled rotational rheometer HAAKE Rheostress 1 (Thermo Fisher Scientific, Waltham, MA). All samples were tested using a cone-plate configuration with a 35 mm diameter and a cone angle of 1°. The protocol used in the measurements was the following: 500 μL of nanogel (1 mg/mL) was pipetted on the lower plate of the rheometer at 25 °C. Then, the upper plate descended until the gap between both plates was that required by the sensor specifications (0.051 mm). A solvent trap was used to avoid nanogel dehydration. After allowing the nanogels to stabilize for 5 min at 25 °C, the oscillatory shear test was executed applying a fixed torque of 5 μN/m at a frequency of 0.1 Hz. The storage *G*′ and loss modulus *G*″ of nanogels were recorded, while the temperature gradually increased from 25 to 50 °C at a constant heating rate.

X-ray diffraction (XRD) was used to assess the crystallinity of BNCs. The diffractograms were recorded in a Philips X’Pert diffractometer with a monochromatized Cu Kα radiation source (λ = 1.54060 Å, 40 kV, 20 mA) with 2θ ranging from 5 to 60° with a step of 0.013 and analysis time of 5 s.

Transmission electron microscopy (TEM) images were recorded in a T20-FEI Tecnai thermionic microscope operated at an acceleration voltage of 200 kV. High-resolution TEM (HRTEM) images were acquired using an FEI Tecnai F30 microscope operated at 300 kV. Samples were dropped in carbon-coated copper grids, dried at room temperature, and stained with a negative staining agent (3% phosphotungstic acid) when necessary to improve the contrast of the polymeric nanoparticles. TEM images were analyzed using the open-source image processing software ImageJ to obtain size distributions of nanoparticles.

### Synthetic Procedures

#### Synthesis of Bupivacaine Free Base

Bupivacaine free base was obtained using the procedure reported by Parshad et al.^57^ with modifications. Bupivacaine hydrochloride (1 g) was dissolved in 50 mL of deionized water; after complete dissolution, its free base form was obtained by adding a 0.2 M NaOH solution dropwise under stirring. The free base form started to precipitate as a white solid, and the NaOH solution was continuously added until a pH of 11 was obtained (p*K*_a_ = 8.4). The resulting white solid was filtered under vacuum and washed with deionized water several times. The solid was dried under vacuum overnight, and its melting point was determined by differential scanning calorimetry (DSC), corroborating the synthesis of free base (m.p. = 107–108 °C) with a 70% yield.

#### Preparation of Bupivacaine Nanocrystals (BNCs)

Bupivacaine nanocrystals (BNCs) were prepared using the antisolvent precipitation technique adapted from methods previously used for other hydrophobic drugs.^25,28^ Bupivacaine free base (50 mg) was dissolved in 1.5 mL of PEG300. The antisolvent solution was prepared by adding 100 mg of the HPMC polymer in 3.5 mL of water. To allow HPMC dissolution in water, the solution was heated at 80 °C under stirring until the polymer powder was well dispersed; subsequently, the polymer solution was cooled down in an ice bath to reach the temperature at which HPMC becomes water-soluble. The drug dissolved in PEG300 was quickly added to the HPMC solution under continuous stirring at 400 rpm and room temperature for 2 min. The final organic and aqueous phase ratio was maintained at 3:7, where HPMC was 2% w/w in the final dispersion. After the addition of drug solution, the clear solution turned into a stable opaque dispersion, indicating the formation of BNCs. To precipitate the BNCs, the final dispersion was centrifuged at 25 000 rpm for 40 min using an ultracentrifuge (Beckman Coulter, Avanti J-20 XP equipped with a JA25.50 rotor), the supernatant was replaced with water, and the solid was redispersed. The BNCs were washed with water to remove the excess of unreacted polymer. Finally, the obtained BNCs were dispersed in 5 mL of deionized water.

#### Synthesis of Bupivacaine Nanocrystal-Loaded Nanogels (BNC-Nanogels)

Thermoresponsive P(MEO_2_MA-*co*-OEGMA_500_) nanogels were prepared using the aqueous precipitation polymerization method by in situ free-radical copolymerization based on the protocol reported by Tian and co-workers,^32^ with several modifications including the use of a different cross-linker, EGDMA. The drug-loaded nanogels were grown introducing the as-prepared BNCs in the nanogel synthesis medium. Briefly, a typical synthesis was performed in a 100 mL Schlenk tube, in which the as-prepared BNCs were mixed with 1.56 mmol of MEO_2_MA monomer, 0.21 mmol of OEGMA_500_ monomer, and 0.06 mmol of EGDMA as a cross-linker. To maintain the lower critical solution temperature (LCST) of the nanogels in the physiological range, we used an [MEO_2_MA]/[OEGMA_500_] monomer molar ratio of 88:12.^33^ SDS surfactant (2 mL, 0.035 mmol) was used as a stabilizer; then, the reagents were dissolved in Milli-Q water, reaching a final volume of 40 mL. The reaction mixture was purged with argon under stirring for 30 min, then the solution was heated to 70 °C, and polymerization was initiated by the addition of KPS (2 mL, 0.037 mmol). The polymerization reaction was carried out for 6 h at 70 °C. The obtained BNC-nanogels were centrifuged at 10 000 rpm (10 min) and 10 °C and washed three times with water to remove unreacted substances. The protocol for the preparation of empty nanogels was identical to the previous one in the absence of BNCs, except for the purification procedure. To recover empty drug-free nanogels after the synthesis, it was necessary to ultracentrifugate them at 25 000 rpm for 60 min at 15 °C (Beckman Coulter, Avanti J-20 XP equipped with JA25.50 rotor) due to the excellent stability of the nanogels in aqueous solution. This makes the separation of empty nanogels from loaded nanogels (that precipitate effectively at 10 000 rpm) possible by repeated washing cycles.

### Bupivacaine *In Vitro* Release

The *in vitro* release profiles of BNCs and BNC-nanogels were determined using 1 mL of 0.180 and 0.196 mg/mL dispersions in water, respectively. The dispersions were selected to have the same drug amount in both samples according to their corresponding drug loading, and to work below the drug saturation concentration. The samples were incubated in a thermoshaker at 37 °C with mechanical agitation at 600 rpm (Biosan TS-100C, Riga, Latvia). For each release time point, three samples were taken out and centrifuged (13 000 rpm, 10 min) and the supernatant was removed and filtered with a 0.22 μm syringe filter. The amount of bupivacaine released at each time was calculated from the supernatant using GC-MS. Each sample (100 μL) was mixed with 850 μL of ethanol and 50 μL of limonene solution in methanol as an internal standard. Drug loading (DL%) in the nanoparticles was determined using GC-MS by extraction of the drug in ethanol. The drug loading was calculated using the following formula
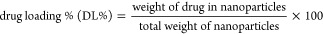


### *In Vitro* Biological Studies

#### Cell Cultures

The *in vitro* effects in cell cultures of the synthesized BNC-nanogels and BNCs were determined at different levels: cell metabolism, cell membrane (apoptosis), and cell nucleus (cell cycle phase distribution). These *in vitro* studies were carried out in human dermal fibroblasts (Lonza, Belgium), the human monocytic cell line THP1 (American Type Culture Collection), and mouse mesenchymal stem cells (mMSCs) and human glioblastoma cells (U251MG), both donated by Dr. Pilar Martín-Duque. Cytotoxicity was evaluated in a battery of different cell lines of different origins (human and murine) to give a general overview of the potential cytotoxicity in different cell lines including somatic (fibroblasts and macrophages), tumoral (U251MG), and multipotent stromal cells (mMSCs). This general cell-based assay represents a screening of different physiological environments, addressing biological activities and potential toxicity issues. Human dermal fibroblasts and U251MG cells were grown in Dulbecco’s modified Eagle’s medium (DMEM) high glucose (Biowest, France), whereas mMSCs were cultured in DMEM-F12 (Biowest, France). Both types of culture media were supplemented with 2 mM l-glutamine (Biowest, France), 10% v/v fetal bovine serum (FBS; Gibco, U.K.), and 1% penicillin–streptomycin–amphotericin B (Biowest, France). On the other hand, THP1 cells (monocytes) were cultured in Roswell Park Memorial Institute (RPMI) 1640 (Biowest, France) supplemented with 2 mM l-glutamine, 10% v/v FBS (Gibco, U.K.), 1% *N*-(2-hydroxyethyl)piperazine-*N*′-ethanesulfonic acid (HEPES), 0.1% 2-mercaptoethanol 50 mM, 1% nonessential amino acids, 1% sodium pyruvate (100 mM), and 1% penicillin–streptomycin–amphotericin B (Biowest, France). THP1 cells were *in vitro* differentiated to macrophages by adding 1 μM phorbol 12-myristate 13-acetate (Sigma-Aldrich) to the cell culture medium for 72 h. All cell types were cultured at 37 °C and 5% CO_2_, except for mMSCs, which were grown under hypoxic conditions (3% O_2_).

#### Subcytotoxic Concentration Determination

The blue cell viability assay (Abnova, Taiwan) was employed to determine the viability related to cell metabolism after cell culture treatment with BNC-nanogels, BNCs, empty nanogels, and bupivacaine hydrochloride solution (0.01–0.5 mg/mL) for 24 h. The reagent was added to the cell cultures (10%) and incubated for 4 h (37 °C, 5% CO_2_). Then, the emitted fluorescence was read (535/590 nm ex/em) in a Synergy HT microplate reader (Biotek). Control samples to evaluate the potential interference of the BNCs and BNC-nanogels in the assays were also assayed. Viability percentages were calculated by the interpolation of the emitted fluorescence from the treated samples and control samples (control samples = 100% viability).

#### Cell Membrane Damage

The potential effects of BNC-nanogels, BNCs, empty nanogels, and bupivacaine hydrochloride treatment in cell membrane were evaluated through the quantitative analysis of cell death by either apoptosis or necrosis by flow cytometry. In brief, cells were treated with these materials at a subcytotoxic concentration determined from the viability assay described above. After 24 h (37 °C, 5% CO_2_), the samples were washed with phosphate-buffered saline (PBS) and collected after centrifugation (1500 rpm, 5 min). Then, the cells were washed and centrifuged again to be further resuspended in 100 μL of Annexin V binding buffer (Annexin V fluorescein isothiocyanate (FITC) kit, Immunostep). Later, the cells were stained in the dark with 50 μL of Annexin V FITC and propidium iodide (15 min, room temperature). Finally, 150 μL of Annexin-binding buffer was added to the samples to be immediately analyzed by flow cytometry (FACSARIA BD equipment and FACSDIVA BD software).

#### Cell Cycle Distribution

The potential changes in the distribution of the cell cycle phases after BNC-nanogels, BNCs, empty nanogels, and bupivacaine hydrochloride solution treatment were studied by flow cytometry. Briefly, the cells were treated with the materials at a subcytotoxic concentration determined from the viability assay described above. After 24 h (37 °C, 5% CO_2_), the cells were harvested in PBS, fixed in 70% ice-cold ethanol, and kept at 4 °C for at least 24 h. Then, DNA staining was carried out through the addition of a solution containing 50 μg/mL propidium iodide and 100 μg/mL RNase A in PBS. Finally, the DNA content was assessed by flow cytometry (FACSARRAY BD equipment and MODIFIT 3.0 Verity software). Control samples were also analyzed to compare the basal status of the cells to that obtained after material treatment.

#### Endotoxin Content Determination

Endotoxin concentration in nanomaterials is required to be sufficiently low for their biomedical application. Endotoxins are lipopolysaccharides (LPS) present in the outer wall of Gram-negative bacteria that can trigger different detrimental effects such as inflammation, immune response, or even the impairment of organ function.^58^ To prevent these harmful effects, the quantification of the endotoxin content in BNCs and BNC-nanogels was determined prior to their application in *in vivo* studies.

The Pierce Chromogenic Endotoxin Quant Kit (Thermo Fisher Scientific) was carried out to quantify the endotoxin content in the different materials (0.1 mg/mL) following the manufacturer’s indications. Briefly, diluted samples (50 μL; 1:100–1:500 in endotoxin-free water) of BNCs and BNC-nanogels were added in a 96-well plate together with the limulus amebocyte lysate (LAL) reagent (37 °C, 10 min). After incubation, the chromogenic substrate solution was added (37 °C, 6 min). Then, the blocking reagent was added and the absorbance was read at 405 nm (Synergy HT microplate reader, Biotek). The endotoxin concentration in BNCs and BNC-nanogels was calculated as indicated by the manufacturer.

### *In Vivo* Sciatic Nerve Blockade

*In vivo* sciatic nerve blockade produced by the different formulations was evaluated using 6–9 weeks old male Sprague–Dawley rats weighing 280–370 g, obtained from Janvier (France). All procedures were carried out under Project License PI01/16 approved by the Ethic Committee for Animal Experiments from the University of Zaragoza. The care and use of animals were performed in accordance with the Spanish Policy for Animal Protection RD53/2013, which meets the European Union Directive 2010/63 on the protection of animals used for experimental and other scientific purposes. The animals were housed in the laboratory animal facilities of the University of Zaragoza. The rats were housed in a conventional animal room (temperature, 20–24 °C), with a relative humidity of 50 ± 5% and a light/dark cycle of 12/12 h. The rats were trained for 1 week before the tests to familiarize them with the experimental procedures (*i.e*., hot plate and weight bearing test) and handling methods, thus diminishing stress-derived effects. Their behavior and the presence of signs of stress were evaluated and scored every day before starting the procedure. Eighteen rats were randomly divided into experimental groups of four animals, except in the case of the control group, in which two animals were used. The animals were initially anesthetized in a chamber with 5% isoflurane under an oxygen flow of 1 L/min and maintained with 2% isoflurane administered *via* a facemask. The right hind limb of each rat was shaved, and the samples were administered through a 21-gauge needle introduced laterally and perpendicularly to the great trochanter upon contact with bone and placed onto the sciatic nerve root. The volume injected was 50 μL containing dispersions of 47 mg/mL BNC-nanogels, 43 mg/mL BNCs, or 40 mg/mL free bupivacaine hydrochloride all having the same equivalent dose of bupivacaine. In this way, in all of the formulations, the total amount of bupivacaine administrated was 2 mg, to compare the anesthetic effect of the nanocrystallization and the nanogel encapsulation. Saline control groups were injected with 50 μL of saline solution, while control groups were not. When the animals recovered from the isoflurane anesthesia, evaluation of the nerve blocking effects of bupivacaine was carried out.

### Evaluation of the Nerve Blocking Efficiency

The local anesthetic effect was assessed through several behavioral tests to evaluate the functional nerve recovery of the animals.^59^ In all of them, the investigators were blinded to the experimental group evaluated. Sensory nerve block was evaluated using a hot plate device; meanwhile, motor nerve block was measured using the weight bearing test. Additionally, two animal reflexes or responses were qualitatively evaluated in the treated paws: paw withdrawal and an adaptation of the extensor postural thrust analysis. Also, the gait ability and the flexion contracture associated with the treated paw position were qualitatively evaluated. Moreover, the rat general behavior, focusing on several qualitative stress signals such as absence of intake or reduced activity, was also daily assessed to monitor the changes and the potential effects of sciatic nerve blocking.

Hot plate test was used to test the nociception or sensory nerve block following previously reported protocols.^60,61^ The intensity of sensory nerve blockade (thermal latency) was measured by placing the plantar surface of the rat’s hind paw on a preheated hot plate (Hot/Cold Plate NG for screening of thermal hyperalgesia/allodynia; Ugo Basile, Italy) at 56 °C. The time required by the animal to remove its foot (thermal latency) was registered using a foot-operated stopwatch. Animals that did not retract the paw after 8 s were removed from the equipment considering a total sensory block to avoid thermal damage in their hind paws. Each measurement was repeated three times using the average of them for the thermal latency analysis. Sensory nerve block was defined as the ratio between the difference in the reaction time observed in one measurement and in the baseline measurement, with respect to the difference between the total blocking time (8 s) and the basal time of each animal.
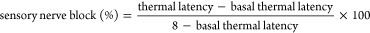
Weight bearing test was performed to evaluate motor nerve blockade following previously reported protocols and correlating with the Randall and Sellito test.^60,61^ Motor block was tested by placing the rat into a holder where the animal is comfortably maintained while its hind paws were placed on two separate sensor weighting plates and letting the animal bear its weight on them (Bioseb Incapacitance Test or Static Weight Bearing (SWB Touch) Test; Bioseb, France). This equipment is based on the equal distribution of weight on both paws in normal rats, whereas the ratio of weight distribution between damaged/impaired and normal paws correlates with the level of discomfort in the rat damaged/impaired paw. Each measurement (10 s) was repeated 10 times using the average of them for the analysis. The motor nerve block was defined as the ratio of the difference between the distribution of weights between the right and left paws at baseline and at the time analyzed, in relation to the said distribution at baseline for each animal. The variable was defined in absolute value since it was observed that the animals with the nerve affected changed their weight distribution toward the right or left leg in the same way.
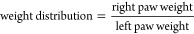

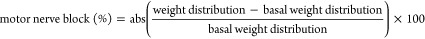
Two animal behavioral reflexes or responses were also tested in the paws: paw withdrawal and an adaptation of the extensor postural thrust analysis. Paw withdrawal represents the animal nociception and defines the move-away reflex of the animal’s paw after mechanical stimulation when tightening it with the operator’s fingers and shows the presence or absence of pain sensitivity among others.^62−64^ It involves the contraction of flexor muscles in the hip, stifle (knee), and hock (ankle).^65−68^ To qualitatively evaluate the responses, values from 1 to 4 were assigned to the observation, where 1 represents a normal withdrawal reflex, 2 a slightly slow reflex, 3 when it only had a slightly delayed reflex, and 4 when there was no reflex. An adaptation of the extensor postural thrust analysis^65,69^ was assessed to study motor function by holding up the rat and placing the two hind limbs extended on a grid and testing if it could hold its body weight and had the reflex of gripping on it with the paw under study. This test evaluates the flexor function of the rat. To qualitatively evaluate the reaction of the animal, values from 1 to 4 were assigned, where 1 represented a normal uphold and grip, 2 to a slight uphold and grip, 3 to a very light uphold and forceless grip, and 4 to the absence of uphold and grip, showing decreased extensor muscle tone and thus a deficit of motor function.^65^

Flexion contracture was assessed following previously reported protocols.^70,71^ Sciatic nerve injuries may cause joint contractures as well as nerve and motor blockage, so the rats use the dorsum of the hurt foot. The lower or less complete reinnervation of the extensor muscles compared to the flexor ones may be the origin of this type of contracture.^71,72^ Therefore, the presence or absence of this contracture was studied to determine the functionality of the sciatic nerve after drug/particles administration. The nonexistence of this contracture was assessed as 0, that is, a fully open stretched foot, and as 1 if the foot showed a flexion contracture.

Gait analysis was assessed adapting previously reported protocols.^73,74^ After nerve injury, the muscles are innervated selectively, so the activation of the muscles during locomotion is abnormal. Therefore, the use of gait analysis provides a noninvasive method to assess the functional status of the sciatic nerve. The animal’s ability to walk was analyzed, assessing this capacity between 1 and 4. It was considered (1) a normal movement and gait of the studied limb, (2) a slightly hindered movement of the limb and gait, (3) a very impaired movement and gait, and (4) total impairment of the gait due to the total lack of movement in the treated hind limb.

Animals were initially tested before anesthetizing them, to know the basal behavior of each of them. After administering the treatment corresponding to their experimental group, the animals were tested, on the first day of treatment, after 0.5, 1, 2, 4, 6, and 8 h after injection. The same parameters were analyzed after 24, 30, 48, 54, 96, and 102 h after administration.

### Histopathological Analysis

All rats were euthanized with carbon dioxide 4 days after injection, and a full postmortem procedure was followed. The sciatic nerve and surrounding tissues were harvested, and all treated rat hind paws were dissected. Moreover, samples from internal organs (*i.e*., gastrointestinal tract, liver, lungs, heart, spleen, and kidneys) were also collected. All samples were fixed in 4% buffered paraformaldehyde, paraffin-embedded, and 5 μm tissue sections were obtained. Slides were routinely stained with hematoxylin and eosin (HE). Additionally, Masson’s trichrome staining was used to elucidate the connective tissue reaction elicited by the administered drug or nanomaterials. Finally, Luxol Fast Blue staining was also performed to stain myelin and better define possible sciatic nerve damage.

### Statistical Analysis

Statistical analysis was performed using ordinary two-way analysis of variance (ANOVA) by means of Prism 7 statistical software (GraphPad Software, San Diego, CA). Statistically significant differences among groups were considered when *p* ≤ 0.05.

## Supporting Information Available

Dynamic temperature sweep of storage modulus *G*′ and loss modulus *G*″ for nanogels (Figure S1); TGA and derivative of TGA curves for (a) bupivacaine hydrochloride and (b) P(MEO_2_MA-*co*-OEGMA_500_) polymer (Figure S2); percentages of both early and late apoptotic, alive, and necrotic cells after treatment with empty drug-free nanogels, BNC-nanogels, BNCs, and bupivacaine hydrochloride for 24 h (Figure S3); cell cycle population distribution (percentage of cells, %) after treatment with empty drug-free nanogels, BNC-nanogels, BNCs, and bupivacaine hydrochloride for 24 h (Figure S4); flow cytometry histograms of cell apoptosis and cell cycle obtained for the control samples; and the samples treated with BNC-nanogels in the four cell lines assayed (Figure S5) (PDF)
