# Supplementary material for: Nanogels with High Loading of Anesthetic Nanocrystals for Extended Duration of Sciatic Nerve Block
Source: ACS Appl Mater Interfaces. 2021 Apr 6;13(15):17220–35. doi: 10.1021/acsami.1c00894 (PMC8892441; doi:10.1021/acsami.1c00894)
Supplement: Supplementary file 1 — am1c00894_si_001.pdf [file am1c00894_si_001.pdf]

SUPPORTING INFORMATION

Nanogels with High Loading of Anesthetic Nanocrystals for Extended Duration of Sciatic Nerve Block

Teresa Alejo[a,b], Laura Uson[a,b], Guillermo Landa[a,b], Martin Prieto[a,b], Cristina Yus Argón[a,b], Sara Garcia-Salinas[a,b], Ricardo de Miguel[c], Ana Rodríguez-Largo[c], Silvia Irusta[a,b,d,e], Victor Sebastian[a,b,d,e], Gracia Mendoza[d,e*], Manuel Arruebo[a,b,d,e]*

[a] Instituto de Nanociencia y Materiales de Aragón (INMA), CSIC-Universidad de Zaragoza, Zaragoza 50009, Spain.

[b] Department of Chemical Engineering, University of Zaragoza, Campus Río Ebro - Edificio I+D, C/ Poeta Mariano Esquillor S/N, 50018 Zaragoza, Spain.

[c] Department of Animal Pathology, Veterinary Faculty, University of Zaragoza, 50013 Zaragoza, Spain

[d] Networking Research Center on Bioengineering, Biomaterials and Nanomedicine, CIBER-BBN, 28029 Madrid, Spain.

[e] Aragon Health Research Institute (IIS Aragón), 50009 Zaragoza, Spain.

* Corresponding authors: Teresa Alejo: teresaal@unizar.es and Gracia Mendoza: gmendoza@iisaragon.es

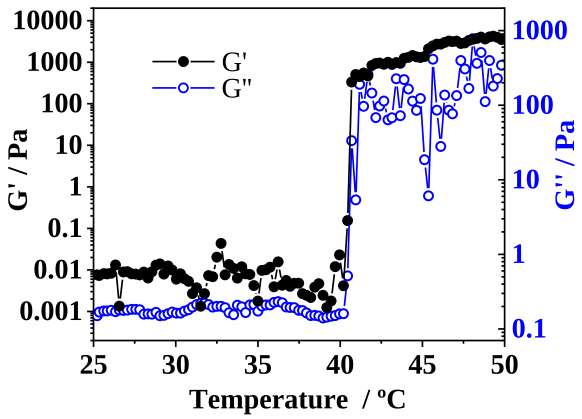

Figure S1. Dynamic temperature sweep of storage modulus G’ and loss modulus G” for nanogels. The rheologically determined volume phase transition temperature is 40.5 ºC.

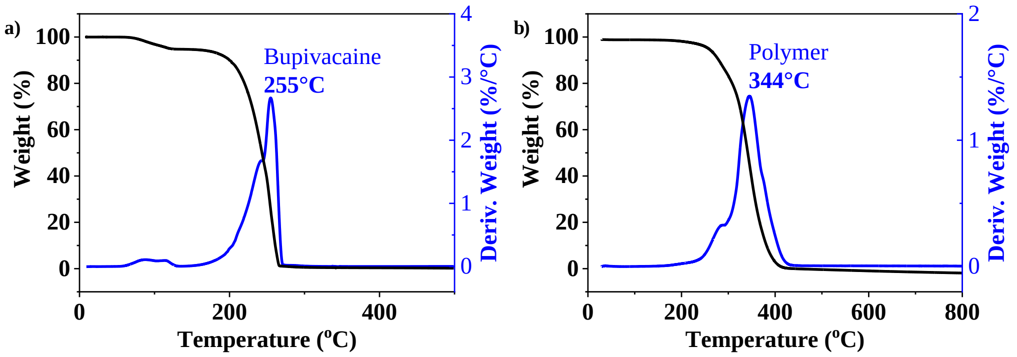

Figure S2. TGA (black) and derivative of TGA (blue) curves for (a) bupivacaine hydrochloride and (b) P(MEO$_2$MA-co-OEGMA$_{500}$) polymer.

Table S1. Mathematical models' correlation coefficients and release exponents for BNC-nanogels and BNCs.

		Nanosystem	
**Kinetic model**	**Parameter**	**BNC-nanogel**	**BNCs**
Zero order	R^2	0.844	0.962
	K_0 (h^{-1})	0.013	0.008
First order	R^2	0.833	0.960
	K (h^{-1})	0.014	0.008
Higuchi	R^2	0.961	0.996
	K_H ($h^{-1/2}$)	0.049	0.027
Korsmeyer-Peppas	R^2	0.998	0.999
	K_{KP} (h^{-n})	0.884	0.963
	n	0.041	0.021
Hixson-Crowell	R^2	0.837	0.961
	K_{HC} (h^{-n})	-0.005	-0.003

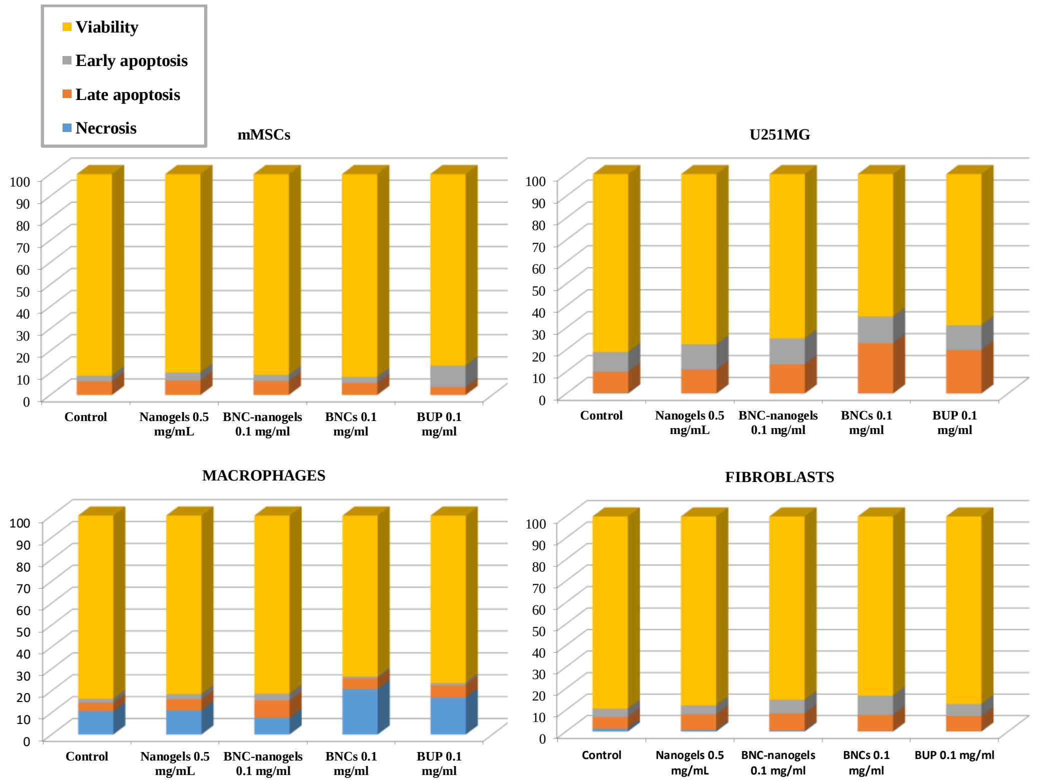

Figure S3. Percentages of both early and late apoptotic, alive and necrotic cells after treatment with empty drug-free nanogels, BNC-nanogels, BNCs, and bupivacaine hydrochloride for 24 h.

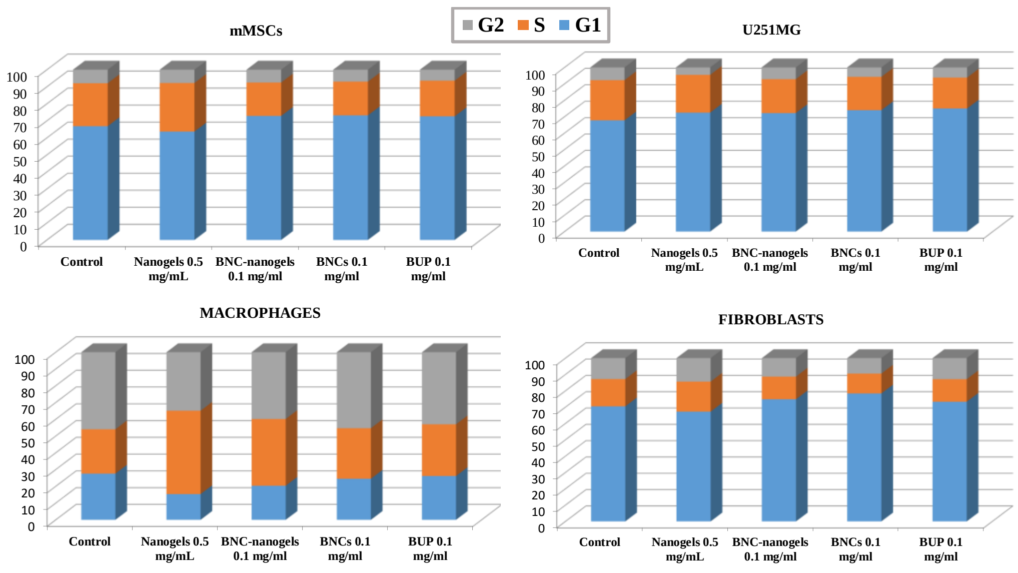

Figure S4. Cell cycle population distribution (percentage of cells, %) after treatment with empty drug-free nanogels, BNC-nanogels, BNCs, and bupivacaine hydrochloride for 24 h.

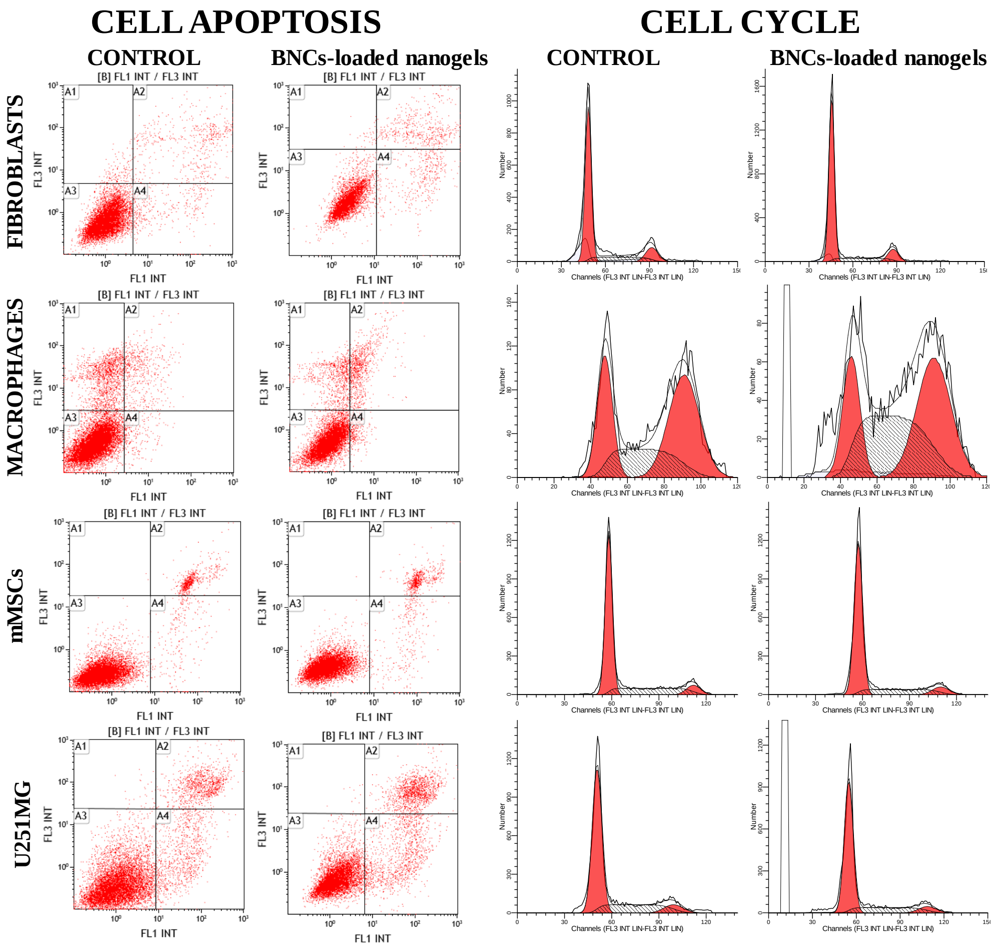

Figure S5. Flow cytometry histograms of cell apoptosis and cell cycle obtained for the control samples and the samples treated with BNC-nanogels in the four cell lines assayed.
